# Detection of toxigenic *Vibrio cholerae* in a traveler with diarrhea lacking classic features, returning from Delhi, India

**DOI:** 10.1016/j.idcr.2026.e02728

**Published:** 2026-08-19

**Authors:** Ryo Mizushima, Masahiro Ishikane, Hidetoshi Nomoto, Masami Kurokawa, Eiji Arakawa, Hidemasa Izumiya, Yukihiro Akeda, Norio Ohmagari

**Affiliations:** a Disease Control and Prevention Center, National Center for Global Health and Medicine, Japan Institute for Health Security, Tokyo, Japan; b Clinical Laboratory, National Center for Global Health and Medicine, Japan Institute for Health Security, Tokyo, Japan; c Department of Bacteriology I, National Institute of Infectious Diseases, Japan Institute for Health Security, Tokyo, Japan

**Keywords:** *Vibrio cholerae*, Traveler's diarrhea, Multiplex PCR

## Abstract

Cholera is an infectious disease caused by *Vibrio cholerae* which has caused several global pandemics since 1817. Currently, Japan is not a cholera-endemic country, and only a few cases of imported disease are reported annually. *Vibrio cholerae* is classified into more than 200 serogroups according to the differences in the O antigen on the cell surface, and only the O1 or O139 groups that produce cholera toxins are called cholera. We report a case in which toxigenic *Vibrio cholerae* O1 was detected in a 64-year-old Japanese traveler returning from Delhi, India, who presented with diarrhea without rice-water stools. Multiple potential diarrheal pathogens were simultaneously detected, including *Clostridioides difficile* toxin A/B and pathogenic *Escherichia coli* (*E. coli*) including enteroaggregative, enteropathogenic, and enterotoxigenic *E. coli*, making definitive attribution of symptoms to a single pathogen difficult by Genetic testing (BioFire FilmArray Gastrointestinal Panel). This case highlights the diagnostic uncertainty that arises with multiplex PCR-based stool testing: while such panels improve detection sensitivity, distinguishing true infection from colonization remains a clinical challenge. Clinicians should integrate laboratory findings with travel history and clinical course rather than relying solely on molecular test results.

## Introduction

Cholera, caused by *Vibrio cholerae*, is an infectious disease that is often indistinguishable from other diarrheal diseases owing to similar symptoms. Historical records suggest its presence in ancient times; however, it gained global attention in 1817 during the first cholera pandemic amid increased transportation and trade [1]. Since then, seven cholera pandemics have been reported in Japan. Japan was notably affected during the third pandemic when cholera entered Nagasaki in 1858 and spread to Edo (presently Tokyo), causing tens of thousands of deaths. While Japan experienced subsequent pandemics, cholera never became endemic, and currently, only a few imported cases are reported annually [2].

*Vibrio cholerae* is classified into over 200 serogroups based on the O antigen, but only the O1 and O139 serogroups that produce cholera toxin cause the disease [3]. *V. cholerae* O1 serogroup has two biotypes, the classical and El Tor biotypes, and two major serotypes, Inaba and Ogawa [4]. The classical biotype was predominant until the sixth pandemic, after which El Tor became dominant in the seventh pandemic starting in 1961 [5]. In 1992, patients with cholera-like diarrhea caused by *V. cholerae* non-O1 serotype (later confirmed as the O139 serotype) suddenly emerged in Chennai, India, and quickly spread throughout India, Bangladesh, and Asia, but it has since declined, with O1 now responsible for nearly all cases [6,7]. Cholera causes approximately 3 million cases and 100,000 deaths annually, with recent outbreaks concentrated in Africa [8]. This report describes a Japanese patient in whom toxigenic *V. cholerae* was detected following travel to Delhi, India.

## Case

The patient was a 64-year-old man with no history of illness or medication use. Three days before the onset, he visited Delhi, India, for business purposes. His business was the design and production of electrical products and he visited India to attend a conference. Before returning to Japan, he had more than 10 watery stools per day (day 0). He returned to Japan without visiting any medical institutions in India. The patient was able to eat and drink; however, the food and drink immediately came out as stools. After returning to Japan, he visited our hospital on day 5 because his condition did not improve.

His symptoms included diarrhea, but he did not have any other symptoms such as fever, skin rash, respiratory symptoms, or joint pain. He had not previously received any vaccines for travel, including cholera. During the trip, he attended meetings and did not work outdoors. The patient had no history of exposure to animals, arthropod bites, or water. As for dietary history, he tried to avoid eating raw food in India but did eat some raw vegetables that were served with fried meat. He had no recent sexual history.

**Fig. 1:**
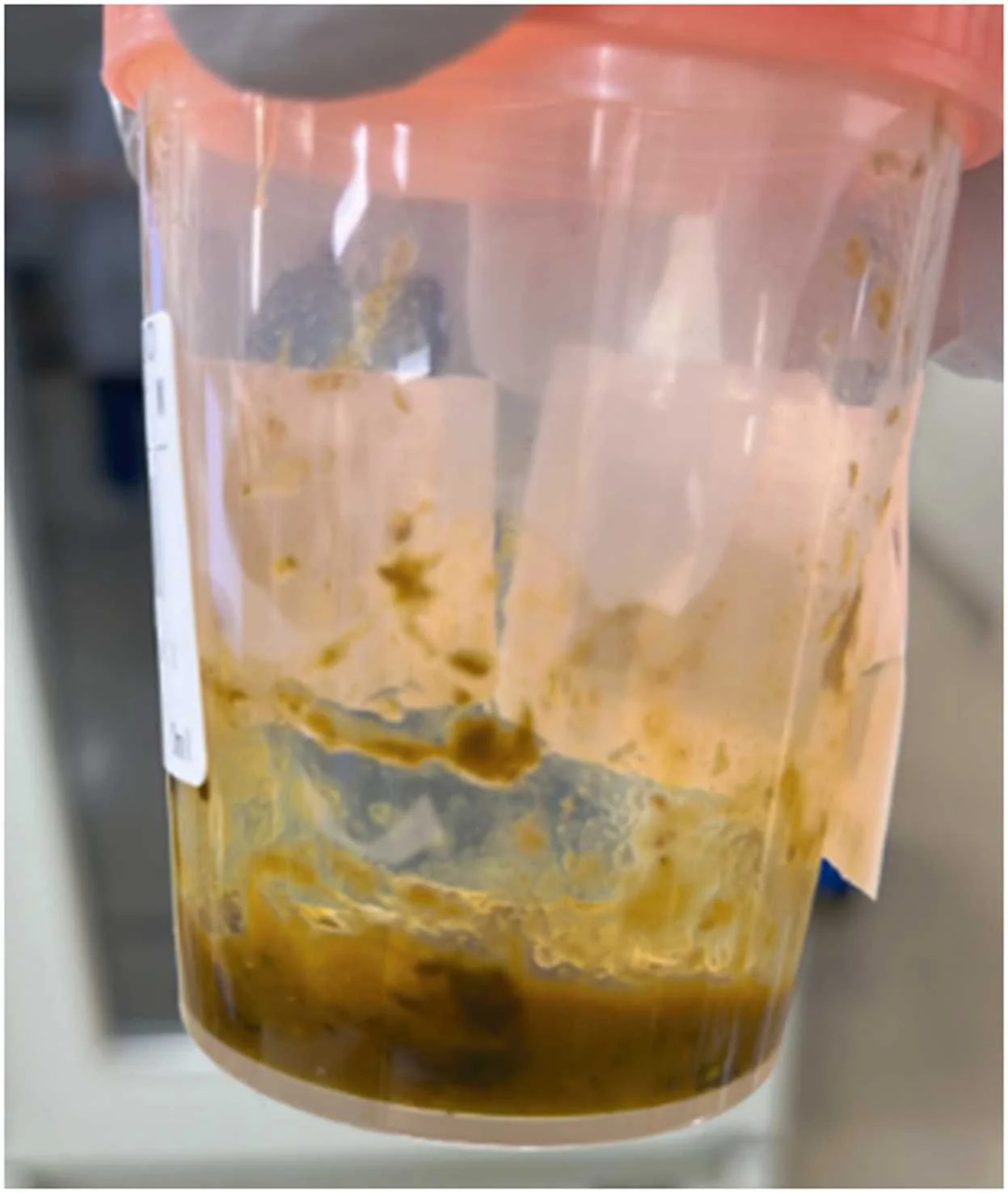
Stool at first visit to our hospital. The stool sample collected at the initial examination was brownish in color, more like soft stool than watery stool, and not like rice-water.

On arrival at our hospital, his vital signs showed a body temperature of 37.3°C, heart rate of 80 bpm, and SpO2 of 98% (ambient air). Physical examination revealed no pale complexion or signs of lethargy with a cholera-like appearance, whereas abdominal examination revealed a flat, soft abdomen with increased abdominal peristalsis. However, no other abnormal findings, such as tenderness, were observed. No abnormalities were observed. Although more than ten watery stools were observed per day, these did not significantly interfere with daily life. The patient was diagnosed with mild diarrhea. Although typhoid fever was considered a differential diagnosis, as there was no fever and the patient's general condition was stable, blood and blood culture tests were not performed, and only a stool culture was conducted. The stool collected at the time of consultation was brownish in color, soft rather than watery, and resembled rice-water (Fig. 1).

Based on the recommendations of the International Society of Travel Medicine (ISTM) guidelines [9], we decided that there was no need for antibacterial drugs or probiotics only. However, as his diarrhea did not improve, he returned to our hospital two days later (day 7). Because there was no significant change in his symptoms, including diarrhea, we prescribed 500 mg azithromycin (AZM) once a day for three days for traveler's diarrhea, based on the ISTM guidelines. The following day, a stool culture revealed *Vibrio*, *Escherichia coli*, and *Enterococcus* species.

Since *Vibrio cholerae* was considered a possible cause of the illness, given his travel history to India, we considered conducting additional tests. However, we did not have the necessary tests to differentiate *Vibrio* based on biochemical properties; therefore, we did not perform these tests. Therefore, a genetic test (BioFire FilmArray Gastrointestinal Panel) was performed on the same day, which yielded positive results for *Vibrio cholerae*. In addition, *Clostridioides difficile* toxin A/B and *Escherichia coli* (enteroaggregative *Escherichia coli* [EAEC], enteropathogenic *Escherichia coli* [EPEC], and enterotoxigenic Escherichia coli [ETEC]) were also positive. The patient was asked to return to our hospital on the same day and cholera treatment was continued with AZM, which had already been prescribed. Considering the possibility of *Clostridioides difficile* infection, we prescribed 125 mg of vancomycin (VCM) orally four times a day for 10 days. Referring to the notification criteria based on the Infectious Diseases Control Law in Japan, we contacted a local public health center to notify them of this case. However, because confirmation of cholera toxin production was necessary for notification, we requested a detailed evaluation from the Department of Bacteriology I of the National Institute of Infectious Diseases in Japan. A detailed investigation revealed that *Vibrio cholerae* belongs to the O1 serogroup, El Tor biotype, and Ogawa serotype. The cholera toxin gene (*ctx*) was detected by PCR [10] and cholera toxin production was confirmed using a VET-RPLA kit (Denka, Japan). Since cholera toxin production was confirmed, notification was accepted by the local public health center. Upon checking the patient's condition, the diarrhea disappeared on day 9, and the patient's general condition improved. During the observation period, there was no single case of rice-water like stool. Also, no secondary cases occurred among family members.

## Discussion

In this case, toxigenic *V. cholerae* O1 El Tor biotype was detected along with multiple other potential pathogens (*Clostridioides difficile* toxin A/B, EAEC, EPEC, and ETEC). As is well recognized, El Tor biotype infection is commonly present without rice-water stools, and the majority of *V. cholerae* O1 infections are mild or asymptomatic.

The incubation period for cholera is reported to be 0.5–4.5 days (median 1.4 days) [11], and the incubation period in our case was within 0–3 days. As the patient was able to drink water orally, we instructed him to drink water properly to avoid dehydration. Regarding antimicrobial agents, AZM, which had already been started as a treatment for travelers’ diarrhea, is also the first-line drug or alternative for cholera; therefore, we continued the administration of AZM and finished the course. AZM was once the drug of choice, but macrolide resistance has widely been reported. In this case, a drug susceptibility test for *Vibrio cholerae* was performed based on the CLSI M45 ED3 (2016); however, the panel (MicroScan Neg Combo EN 2 T) used did not include susceptibility testing for AZM, and therefore the susceptibility test result for AZM in this case remains unknown. The detected *Vibrio cholerae* belonged to the O1 subgroup, El tor biotype, and Ogawa serotype, and the same type was prevalent in the past Indian epidemic, so it matched this case [12].

After the diagnosis of cholera, the patient's eating history was rechecked, and he was found to eat out at restaurants at night. He had been trying to avoid eating raw food; however, at the restaurant, he had been eating cut fruits and fruit juices. One of the local staff members who attended the meeting complained of feeling unwell; although the symptoms were unknown, his condition improved. Although the clear infection route in our case was unknown, he might have been infected through the cut fruit he ate at dinner or through his colleague who felt unwell. However, none of the people who attended meetings or dinners with him complained about feeling unwell.

Cholera diarrhea is classically, characterized by “rice-water like” and white or grayish-white watery stools. In these patients, the loss of body fluids can quickly lead to dehydration and shock [13]. However, most patients with cholera have mild or no symptoms, and many cholera patients do not have rice-water stools, especially in those infected with *V. cholerae* O−1 biotype El Tor [14]. Approximately 1 in 10 people develop severe symptoms, such as watery diarrhea, vomiting, and leg cramps. Since multiple potential pathogens (*Clostridioides difficile* toxin A/B and EAEC, EPEC, and ETEC) were detected simultaneously, *Vibrio cholerae* could not be excluded as a colonizer, and the possibility that symptoms were attributable to other detected pathogens could not be ruled out.

Each of these pathogens acts on the intestinal epithelium through distinct mechanisms: cholera toxin binds to GM1 ganglioside and increases cAMP levels; *Clostridioides difficile* toxin A/B causes cytoskeletal disruption by inactivating Rho GTPase; EAEC causes mucosal adhesion via aggregative adherence fimbriae (AAF) and induces IL-8; EPEC impairs epithelial barrier function via the type III secretion system (TTSS) by forming attaching and effacing (A/E) lesions; and ETEC promotes intestinal fluid secretion via STa/LT. Since the sites of action of each toxin differ, symptoms may be additively or synergistically worsened or modified in cases of co-infection. The incubation period for diarrhea caused by other pathogens detected in this case was similar to that for cholera; hence, it was difficult to identify the main causative microorganism in this case.

Although there have been large cholera outbreaks in Yemen in recent years [15], the main areas of recent cholera outbreaks have been in Africa, and the number of reports from India is relatively low. According to a report by the World Health Organization (WHO), 66,700 cases of cholera will be reported worldwide in 2023, of which 2044 will be reported in India (as of December 15, 2023) [16]. In addition, Delhi is a region with relatively few previous reports [12]. Our case occurred in May, but according to previous reports, outbreaks tend to occur more frequently during the monsoon season from June to September [12]; therefore, the timing of our case fell outside the peak transmission season. Although the number of reports from India is small, there is a high possibility that the number of reports is underestimated, and problems with the surveillance system in India have been identified as the reason for this. Specifically, problems with the testing system, reporting system for diagnoses, evaluation of outbreaks to prevent further infection, and the lack of an early intervention system have been pointed out [17]. In fact, there was a cholera outbreak in Delhi associated with drinking untreated city water [18]. It cannot be ruled out that Delhi is not a cholera endemic area. Our case highlights the importance of considering toxigenic *V. cholerae* in the differential diagnosis of traveler's diarrhea and underscores the diagnostic uncertainty inherent in multiplex PCR-based testing when multiple pathogens are detected simultaneously.

With the widespread multiplex PCR for stool samples, it may become possible to detect atypical and mild cases of cholera that were previously overlooked. While this will redefine the spectrum of causes of traveler’s diarrhea and improve diagnostic accuracy, it also raises the issue of distinguishing between colonization and infection, which could potentially lead to the provision of excessive medical care in some cases. Therefore, clinicians must not rely solely on laboratory test results but must base their clinical management on comprehensive information, including the patient’s travel history and clinical course.

In conclusion, we report a case in which toxigenic *Vibrio cholerae* was detected which may have been overlooked because it is difficult to conduct a detailed examination of *Vibrio* species depending on the testing systems of medical institutions. The widespread use of multiplex PCR for stool samples raises the issue of distinguishing between colonization and infection, which could potentially lead to overtreatment in some cases. Clinicians must not rely solely on laboratory test results, but should base their clinical management on comprehensive information, including the patient's travel history and clinical course.

## CRediT authorship contribution statement

**Ryo Mizushima:** Writing – original draft, Data curation. **Masahiro Ishikane:** Writing – original draft, Project administration, Investigation, Funding acquisition, Data curation. **Hidetoshi Nomoto:** Writing – original draft, Project administration. **Masami Kurokawa:** Writing – original draft, Methodology. **Eiji Arakawa:** Writing – original draft, Methodology. **Hidemasa Izumiya:** Writing – original draft, Methodology. **Yukihiro Akeda:** Writing – review & editing. **Norio Ohmagari:** Writing – review & editing.

## Authorship statement

RM, MI, and HN designed the study, obtained clinical samples, contributed to data collection and verification, and wrote the manuscript. MK, EA, and HI conducted microbiological analyses and wrote the manuscript. YA and NO reviewed the study design and manuscript. All members contributed to the management and administration of the trials. All the authors met the ICMJE authorship criteria.

## Ethics declaration

Written informed consent to take part in the study and to publish the article has been obtained from all participants or their legal representatives. The privacy rights of participants have been observed.

This study was performed in compliance with relevant laws, regulatory frameworks and guidelines where the research took place. Ethics committee approval was not required under relevant laws and institutional guidelines. Institutional review board approval was not required for this case report in accordance with institutional policy and national ethical guidelines. Written informed consent was obtained from the patient for publication of this manuscript.

This research follows the CARE guidelines and the CARE checklist.

## Ethical approval

The patient provided consent for publication of his clinical case details.

## Funding source

This research was supported by 10.13039/100009619AMED [grant number JP24fk0108683]. The funders played no role in the study design; collection, analysis, and interpretation of data; writing of the report; or decision to submit the article for publication.

## Declaration of Competing Interest

The authors declare the following financial interests/personal relationships which may be considered as potential competing interests: Masahiro ISHIKANE reports financial support was provided by AMED. If there are other authors, they declare that they have no known competing financial interests or personal relationships that could have appeared to influence the work reported in this paper.
